# miR‐126‐3p is essential for CXCL12‐induced angiogenesis

**DOI:** 10.1111/jcmm.16460

**Published:** 2021-06-12

**Authors:** Kévin Bassand, Laurent Metzinger, Meriem Naïm, Nesrine Mouhoubi, Oualid Haddad, Vincent Assoun, Naïma Zaïdi, Odile Sainte‐Catherine, Amena Butt, Erwan Guyot, Olivier Oudar, Christelle Laguillier‐Morizot, Angela Sutton, Nathalie Charnaux, Valérie Metzinger‐Le Meuth, Hanna Hlawaty

**Affiliations:** ^1^ INSERM U1148, Laboratory for Vascular Translational Sciences (LVTS), UFR SMBH Université Sorbonne Paris Nord Bobigny France; ^2^ HEMATIM UR 4666, Centre Universitaire de Recherche en Santé (CURS), Université de Picardie Jules Verne, CHU‐Amiens‐Picardie Amiens France; ^3^ Laboratoire de Biochimie Hôpital Avicenne Assistance Publique‐Hôpitaux de Paris Bobigny France

**Keywords:** angiogenesis, chemokine CXCL12, endothelial cells, miR‐126

## Abstract

Atherosclerosis, in the ultimate stage of cardiovascular diseases, causes an obstruction of vessels leading to ischemia and finally to necrosis. To restore vascularization and tissue regeneration, stimulation of angiogenesis is necessary. Chemokines and microRNAs (miR) were studied as pro‐angiogenic agents. We analysed the miR‐126/CXCL12 axis and compared impacts of both miR‐126‐3p and miR‐126‐5p strands effects in CXCL12‐induced angiogenesis. Indeed, the two strands of miR‐126 were previously shown to be active but were never compared together in the same experimental conditions regarding their differential functions in angiogenesis. In this study, we analysed the 2D‐angiogenesis and the migration assays in HUVEC in vitro and in rat's aortic rings ex vivo, both transfected with premiR‐126‐3p/‐5p or antimiR‐126‐3p/‐5p strands and stimulated with CXCL12. First, we showed that CXCL12 had pro‐angiogenic effects in vitro and ex vivo associated with overexpression of miR‐126‐3p in HUVEC and rat's aortas. Second, we showed that 2D‐angiogenesis and migration induced by CXCL12 was abolished in vitro and ex vivo after miR‐126‐3p inhibition. Finally, we observed that SPRED‐1 (one of miR‐126‐3p targets) was inhibited after CXCL12 treatment in HUVEC leading to improvement of CXCL12 pro‐angiogenic potential in vitro. Our results proved for the first time: 1‐the role of CXCL12 in modulation of miR‐126 expression; 2‐the involvement of miR‐126 in CXCL12 pro‐angiogenic effects; 3‐the involvement of SPRED‐1 in angiogenesis induced by miR‐126/CXCL12 axis.

## 1 INTRODUCTION

Atherosclerosis is a vascular pathology leading to the partial or the total obstruction of the blood vessels and flow perturbation, the decrease in oxygen and nutrients leading to ischemia, cell death and finally to the tissue necrosis. To prevent the necrosis and induce tissue regeneration, angiogenesis needs to be initiated. This physiological process, which involves endothelial cells (EC) response to several stimuli, consists of cell proliferation, migration and cell‐to‐cell interactions to form functional vessels. In an ischemic tissue, the oxygen decrease allows the synthesis of pro‐angiogenic factors secreted by adjacent EC in order to stimulate angiogenesis. Among these factors, Vascular Endothelial Growth Factor (VEGF)‐A and Fibroblast Growth Factor (FGF)‐2 bind to their tyrosine kinase receptors to induce intracellular signalling pathways involved in angiogenesis.^1^ The chemokines are the small soluble proteins belonging to the family of chemoattractant cytokines that are secreted in ischemic areas.^2^, ^3^ Interestingly, we and others have already shown that chemokines can also be involved in angiogenesis.^4^, ^5^ CXCL12 (SDF‐1α) presence in the ischemic tissue allows for the recruitment of endothelial progenitor cells leading to local reendothelialization.^6^ In addition, CXCL12 stimulates angiogenesis by binding to their specific seven transmembrane domains receptors coupled to G proteins, such as CXCR4 or CXCR7. This binding induces the intracellular signalling pathways involved in angiogenesis such as the MAPK Erk1/2.^7^ This phenomenon can also be stimulated by modulation of microRNA (miRs) expression.^8^ miRs are small, single‐stranded, non‐coding RNAs involved in the regulation of gene expression. By binding to their specific targeted mRNAs, they can induce their total degradation or repress their protein translation.^9^ Over the last few years, miRs have been extensively studied in the context of angiogenic processes. Indeed, three classes of miRs could be dissociated: pro‐angiogenic miRs, anti‐angiogenic miRs and miRs with a dual role.^10^ Among them, miR‐126, strongly expressed in EC, has been found to be implicated in angiogenesis.^11^, ^12^ miR strand selection determines which one of the two strands (−5p or −3p) becomes the active strand, and this varies according to cell type and disease state.^13^ Interestingly, it was previously shown that both strands of miR can be functional and have different targets.^14^, ^15^, ^16^ In some miR species, including miR‐126, both the passenger strand (miR‐126‐5p) and guide strand (miR‐126‐3p) have been shown to improve the biological effects, complicating the interpretation of their action.^16^ The pro‐angiogenic role of miR‐126‐3p has been extensively studied. Indeed, it is implicated in the Erk1/2 signalling pathway induced by VEGF‐A through a repression of the protein SPRED‐1^17^ whose role is to inhibit the expression of the small protein G Ras.^18^ Although mainly degraded, miR‐126‐5p has also demonstrated its pro‐angiogenic role in vitro, but also in vivo by reduction of intimal hyperplasia and by stimulation of EC proliferation.^19^ In EC, the link between miR‐126‐3p and chemokine CXCL12 has been already demonstrated.^20^ Indeed, we have previously shown that overexpression of miR‐126 led to an increase of CXCR4 protein expression, a CXCL12’s receptor.^21^ Finally, overexpression of miR‐126 caused an increase of CXCL12 synthesis by EC.^20^

The originality and interest of our study was to compare in the same experimental conditions the effect of both miR‐126‐3p and −5p strands in two angiogenesis models, in vitro and ex vivo. Also, our aim was to determine whether the modulation of their expression (miR‐126‐3p and −5p) is involved in the vascular tube formation induced by CXCL12.

## 2 MATERIALS AND METHODS

### 2.1 Cell culture

Human Umbilical Vein Endothelial Cells: HUVEC (ATCC^®^ CRL‐1730TM) were cultured in Endothelial Cell basal medium 2 (ECBM2, ref C22211, Promocell) and supplemented with 12% of Foetal Bovine Serum (to induce cell division every 16 hours), 5 ng.mL^‐1^ Epidermal Growth Factor, 0.2 μg.mL^‐1^ hydrocortisone, 0.5 ng.mL^‐1^ VEGF, 10 ng.mL^‐1^ bFGF, 20 ng.mL^‐1^ Insulin like Growth Factor, 1 μg.mL^‐1^ ascorbic acid and 100 Units.mL^‐1^ of penicillin and 100 µg.mL^‐1^ of streptomycin. The cells were cultured in an incubator at 37°C under a controlled atmosphere of 5% CO_2_.

The Huh7 human hepatoma cell line was grown in Dulbecco's minimal essential medium supplemented with glucose (1 g.L^‐1^), 10% of Foetal Bovine Serum, streptomycin (100 UI.mL^‐1^) and penicillin (100 UI.mL^‐1^) (Invitrogen). Cells were grown at 37°C in disposable plastic flasks, in a humidified atmosphere containing 5% CO_2_. The medium was replaced twice weekly, and cells were trypsinized and diluted every 3 days at a ratio of 1:3.

### 2.2 Animal model

Aortas from euthanized Sprague‐Dawley rats (Janvier Labs) were harvested. Animals were anesthetized by intraperitoneal pentobarbital injection (60 mg/kg) and sacrificed by abdominal artery section. Experimental protocol was realized in accordance with the European Communities Council Directive of September 22, 2010 (2010/63/EU) for animal care. Experiments were performed in Université Sorbonne Paris Nord (Bobigny, FRANCE, agreement number A 9300801). Tissues were recovered from euthanized animals obtained from the laboratory of Pr‐ Carole PLANES (INSERM U1272, Université Sorbonne Paris Nord), whose research protocol was approved by the institutional reviewing with animal experimentation and accorded with animal welfare guidelines (Ministère Français de l’Enseignement Supérieur, de la Recherche et de l’Innovation, Paris, FRANCE) (CEEA – 005; Comité d’éthique en expérimentation animale Charles Darwin; C2EA‐06, authorization C9300801, authorization APAFIS #8150, approved on august 2017, the 8th).

### 2.3 Western blot

For the SPRED‐1 protein expression analysis, 20 μg of total proteins were loaded on a 7% poly‐acrylamide gel and then transferred to a nitrocellulose membrane (ref 10600001, GE Healthcare). The membranes were saturated twice 1‐hour with baths containing TBS/T (TBS, 0.1% Tween 20) and 5% milk. An anti‐SPRED‐1 (E‐5) antibody (sc‐393198, Santa Cruz) was added diluted to 1/500e in TBS/T and 5% milk overnight. The detection was made by incubation with a secondary goat anti‐mouse antibody diluted to 1/2000e (ref P044701, DAKO) for 1 hour and then by adding the ECL solution (Pierce^®^ ECL Western Blotting Substrate #32106) and digital reading using Chemidoc apparatus and the Image Lab 4.2 software (Bio‐Rad).

### 2.4 Transfection

HUVEC were transfected with premiR‐126‐3p (Assay ID PM2841, Fisher Scientific), premiR‐126‐5p (Assay ID PM10401, Fisher Scientific), antimiR‐126‐3p (Assay ID AM10401, Fisher Scientific), antimiR‐126‐5p (Assay ID AM12841, Fisher Scientific) or scramble negative control (SCL) at the concentration of 20 nmol.L^‐1^ in the presence of INTERFERin transfection reagent according to the manufacturer's instructions (Polyplus) and incubated for 24 hours at 37°C with 5% CO_2_. Cells were then harvested for further analysis.

### 2.5 qRT‐PCR

Total RNAs from transfected or not transfected HUVEC were isolated using the RNeasy^®^ Plus minikit (QIAGEN) according to the manufacturer's instructions. The purity of total RNAs was analysed by measuring the 260/280 and 260/230 nm optical density ratios. Reverse Transcription was performed using 1μg of total RNA using High Capacity cDNA Synthesis Kit (Applied Biosystem) according to the manufacturer's instructions. For reverse transcription of miR‐126‐3p, miR‐126‐5p or U6 small nuclear RNA (used as an endogenous control for miR expression), specific RT primers were added to the master mix. Finally, PCR reactions were performed with Taqman Universal Master Mix (Applied Biosystem) using the following Taqman primers: miR‐126‐3p (Hsa‐miR‐126‐3p‐Assay ID 002228, Fisher Scientific), miR‐126‐5p (Hsa‐miR‐126‐5p‐Assay ID 000451, Fisher Scientific), U6 (U6snRNA‐Assay ID 001973, Fisher Scientific).

### 2.6 Aortic ring assay

To study the role of miR‐126 and CXCL12 in ex vivo angiogenesis, aortas were collected from 5 weeks old Sprague‐Dawley rats, fragmented into 1 mm rings and put on a layer of Matrigel (ref.354248, Corning) and then cultured in complete ECBM2 medium containing 2% of FBS (ref.C22211, Promocell) for 48 hours. These rings were transfected or not by premiR‐126 or antimiR‐126 (20 nmol.L^‐1^) for respectively the miR‐126‐3p or miR‐126‐5p species for 48 hours and stimulated or not by CXCL12 at 6 nmol.L^‐1^ (ref.350‐NS‐050, R&D system) for 96 hours (2 × 48 hours). Finally, the aortic rings were fixed with 4% Paraformaldehyde and photographed under phase contrast microscope to quantify the total coverage area (in mm^2^), the quantity of meshes (N) and finally the maximum distance of migration (in mm).

### 2.7 2D‐angiogenesis

To study the role of miR‐126 and CXCL12 in vascular tube formation in vitro, HUVEC were transfected for 18 hours by premiRs‐126 or antimiRs‐126 (20 nmol.L^‐1^) for respectively the miR‐126‐3p or miR‐126‐5p strands. The cells were removed using PBS/EDTA (10mmol.L^‐1^) and 7000 cells were deposited on a thin layer of Matrigel (ref. 354248, Corning) pre‐casted in 96‐well plates and incubated for 6 hours at 37°C, 5% CO_2_, stimulated or not by CXCL12 at 6 nmol.L^‐1^. The results present quantification of the number of meshes carried out under a phase contrast microscope by mapping over an entire well using the Archimed^(TM)^ and Histolab^(TM)^ software. The representation of the whole well was done using the reconstitution of the different fields. The fields were analysed with the Cartograph module of the Archimed^(TM)^ software. The multifocus module allowed us to acquire images on different focus planes in order to obtain the clear image of the whole well. The number of meshes represented by closed areas surrounded by segments and associated junctions formed by HUVECs was quantified by manual tracing using the Histolab ^(TM)^ software.

### 2.8 Migration assay

HUVEC migration was studied using a modified Boyden Chamber. First, the upper chamber was precoated with fibronectin (100 µg.mL^‐1^) overnight at 4°C. After removing the excess of fibronectin, chambers were saturated with DMEM containing 0.1% BSA for 30 minutes at 37°C, 5% CO_2_. Then, 5.10^4^ HUVEC transfected or not were deposited on the upper chamber containing 500 μL of complete ECBM2 medium supplemented with 12% of FBS. Migration was stimulated by adding 1 mL of complete medium with or without CXCL12 at 6 nmol/L during 24 hours. At the end of the experiment, cells were fixed using 4% of paraformaldehyde, coloured using haematoxylin‐Hemalum and quantification of migrated cells was performed under phase contrast microscope.

### 2.9 Luciferase assay

To study the effect of CXCL12 on egfl7‐miR‐126 promoter activity, Huh7 cells were cotransfected with plasmid pGL3Basic‐miR‐126‐EGFL7‐Promoter (Addgene) and control pGL4.73 [hRluc/SV40] (Promega) for 24 hours. Then, the cells were stimulated or not by CXCL12 for 24 hours at 6 nmol.L^‐1^. Detection of luminescence was performed using Dual‐Glo^®^ Luciferase Assay System (Promega) following the manufacturer's instructions.

### 2.10 Statistical analysis

All the results are presented with mean ± SEM. For statistical analysis, non‐parametric tests were performed using GraphPad Prism software. Independent sample *t* tests (Mann and Whitney) were applied to compare two groups when the data followed a normal distribution and one‐way analysis of variance (ANOVA) was used to compare among several groups. *p* <.05 indicated statistically significant differences.

## 3 RESULTS

### 3.1 CXCL12‐induced miR‐126 expression in vitro and ex vivo

**FIGURE 1:**
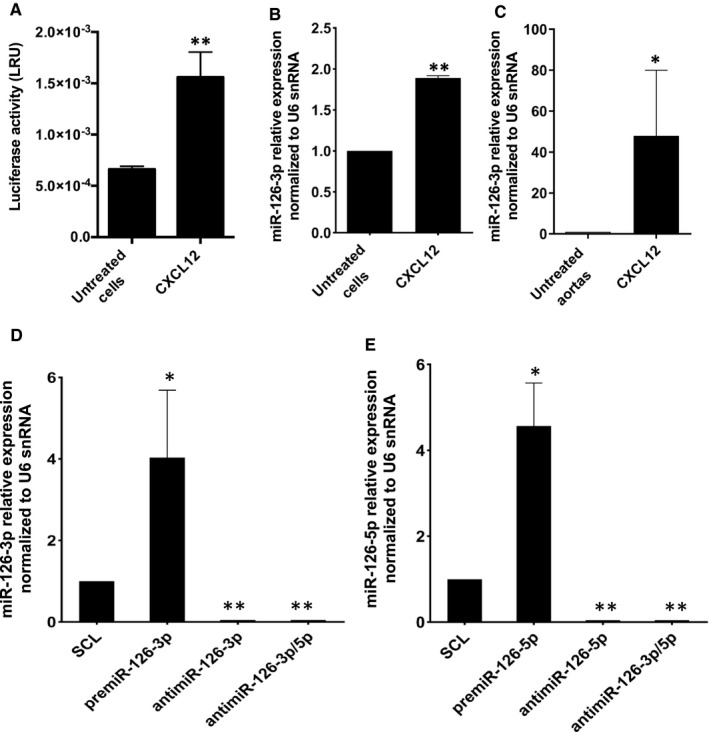
**CXCL12‐induced miR‐126‐3p endogenous expression in vitro and ex vivo**. (A) To study the effect of CXCL12 on egfl7‐miR‐126 promotor activity, Huh7 were co‐transfected with plasmid pGL3Basic‐miR‐126‐EGFL7‐Promoter (Addgene) and control pGL4.73 [hRluc/SV40] (Promega) for 24 hours. Then the cells were stimulated or not stimulated by CXCL12 for 24 h at 6 nmol.L^‐1^. Detection of luminescence was performed using Dual‐Glo^®^ Luciferase Assay System (Promega). To study the effect of CXCL12 on miR‐126 expression level, HUVEC (B) or rat aortas (C) were stimulated or not stimulated by CXCL12 for 24 h at 6 nmol.L^‐1^. After total RNA extraction, miR‐126 level expression was analysed using qRT‐PCR with U6 snRNA as endogenous control. The results are expressed as mean ± SEM. Three independent experiments were performed for in vitro experiments and six for ex vivo experiments. **p <.01 vs Untreated cells; *p <.05 vs Untreated aortas. To analyse the up and down regulation of miR‐126‐3p and miR‐126‐5p, HUVEC were transfected with 20 nmol.L^‐1^ of premiR‐126‐3p, premiR‐126‐5p or inhibitors for 24 h. After total RNA extraction, miR‐126‐3p (D) and miR‐126‐5p (E) expression levels were analysed performing qRT‐PCR using U6 snRNA as endogenous control. The results are expressed with mean ± SEM. Three independent experiments were performed. **p <.01 vs SCL; *p <.05 vs SCL.

We and others showed that miR‐126‐3p regulates CXCL12 expression. Knowing that miR‐126 is encoded by the *egfl7* gene, and in order to study a potential reverse effect of CXCL12 on miR‐126 expression, we first decided to study the *egfl7* promoter activity. The results showed (Figure 1A) that there was a significant increase of 2.35 ± 0.35‐fold of promoter activity after stimulation by CXCL12 as compared to untreated cells. To confirm this result, we decided to analyse the miR‐126‐3p expression in HUVEC and in rat aortas ex vivo after CXCL12 (6 nmol.L^‐1^) stimulation for 24 hours. The results showed that there was a significant increase of miR‐126‐3p level up to 88 ± 5% when HUVEC were stimulated with CXCL12 as compared to untreated cells (Figure 1B). In addition, in our ex vivo model, there was a significant increase up to 48 ± 32‐fold after CXCL12 stimulation as compared to untreated aortas (Figure 1C).

Then, we studied the implications of both strands of miR‐126 (miR‐126‐5p and miR‐126‐3p) in angiogenesis processes induced by CXCL12. First, we set up the conditions needed to up‐regulate (premiR) and down‐regulate (antimiR) both miRs strands in HUVEC. The results showed that there was a significant increase of miR‐126‐3p level up to 4 ± 1.2‐fold after premiR‐126‐3p transfection as compared to scramble negative control (SCL) (Figure 1D).

Moreover there was an abolition of miR‐126‐3p level after antimiR‐126‐3p transfection. The transfection with both antimiR‐126‐3p and antimiR‐126‐5p (antimiR‐3p/5p) induced the same results. In parallel, there was a significant increase of miR‐126‐5p level up to 4.6 ± 0.8‐fold after premiR‐126‐5p transfection as compared to SCL. In addition, miR‐126‐5p expression was also extinguished after antimiR‐126‐5p or both antimiR‐126‐3p/5p transfection (Figure 1E). To control the cell viability after the transfection with all miRs, the MTT tests were performed. The results showed (*Data not shown*) that there was no toxicity after any miRs transfection as compared to SCL control.

Our results demonstrated that CXCL12 enhanced miR‐126 expression and we validated the up‐ and down‐regulation of the various miRs species.

The second step of this work was to study the role of these miRs on HUVEC migration and two‐dimensional (2D) angiogenesis test.

### 3.2 miR‐126‐3p increased HUVEC migration and 2D‐angiogenesis in vitro

**FIGURE 2:**
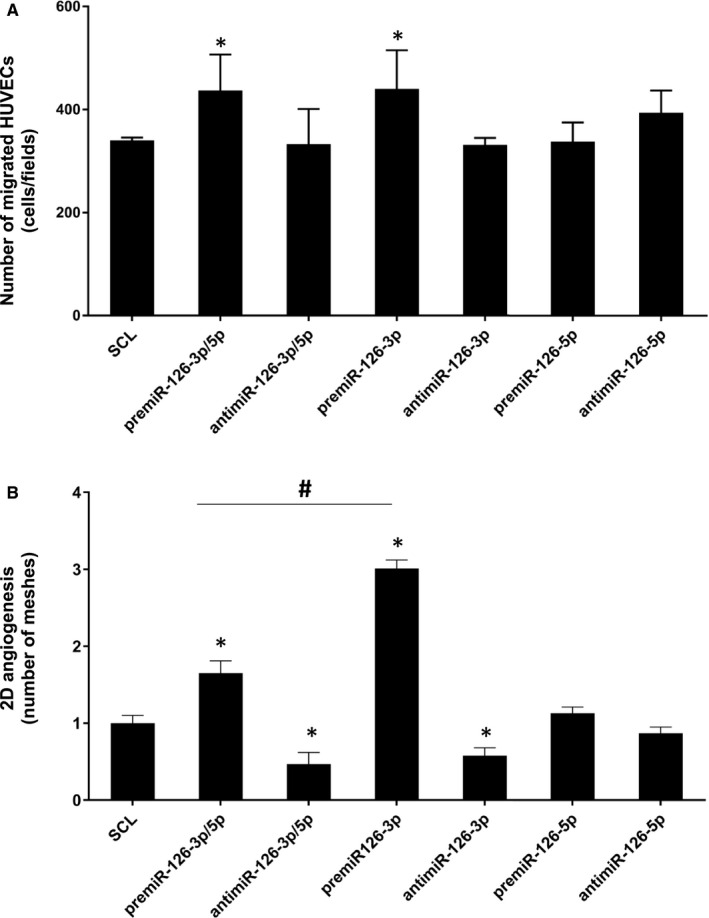
**Effect of miR‐126‐3p and miR‐126‐5p on HUVEC migration and vascular tubes formation**. The miR‐126 was up‐ or down‐regulated by transfecting HUVEC with 20 nmol.L^‐1^ of premiR‐126 or inhibitors for 24 h. (A) Migration assay was performed using Boyden chamber. 5.10^4^ transfected cells were seeded on the upper compartment during 24 h; the number of migrated cells was determined using phase contrast microscope. (B) Vascular tubes formation in 2D on Matrigel. 7500 transfected cells were deposited on the top of Matrigel and the tubular formation was studied after 6 h of incubation. The quantity of meshes was determined using phase contrast microscope and Archimed^(TM)^ and Histolab^(TM)^ software. For each assay, three independent experiments were performed. *p <.05 vs SCL; ^#^ *p* <.01 premiR‐126‐3p/5p vs premiR‐126‐3p

Our results showed that there was a significant increase of HUVEC migration up to 29.4 ± 16% and up to 28.5 ± 15% after premiR‐126‐3p alone or both premiR‐126‐3p and premiR‐5p (premiR‐126‐3p/5p) transfection respectively (Figure 2A) as compared to SCL. However, there was no effect on cell migration after premiR‐126‐5p alone, antimiR‐126‐3p alone or both antimiR‐126‐3p/5p transfection as compared to SCL.

Then, we studied the effect of the various miRs treatments on 2D‐angiogenesis by analysing the number of meshes formed by HUVEC on Matrigel layer. The most striking result was a significant increase of the number of meshes up to 3 ± 0.1 fold after premiR‐126‐3p transfection as compared to SCL (Figure 2B). In contrast, there was no significant effect after transfection of premiR‐126‐5p only. The transfection of both premiR‐126‐3p/5p gave an intermediary result with a significant increase of the number of meshes up to 65 ± 16%. However, this result was significantly lower than with transfection of premiR‐126‐3p only, suggesting an inhibitory effect of miR‐126‐5p on miR‐126‐3p pro‐angiogenic action. In the case of the inhibitory treatments, there was a significant decrease of 2D‐angiogenesis up to 42 ± 10% after transfection of antimiR‐126‐3p only as compared to SCL. Interestingly, the transfection with both antimiR‐126‐3p/5p led to a significant decrease of 53 ± 15% as compared to SCL. In contrast, there wasn't any effect on 2D‐angiogenesis after transfection of antimiR‐126‐5p only.

Taken together, our data suggest that miR‐126‐3p/5p and miR‐126‐3p act as positive regulators of HUVEC migration and vascular tubes formation, but miR‐126‐5p did not seem to have any effect on this physiological process.

### 3.3 miR‐126‐3p was implicated in CXCL12‐induced HUVEC migration

**FIGURE 3:**
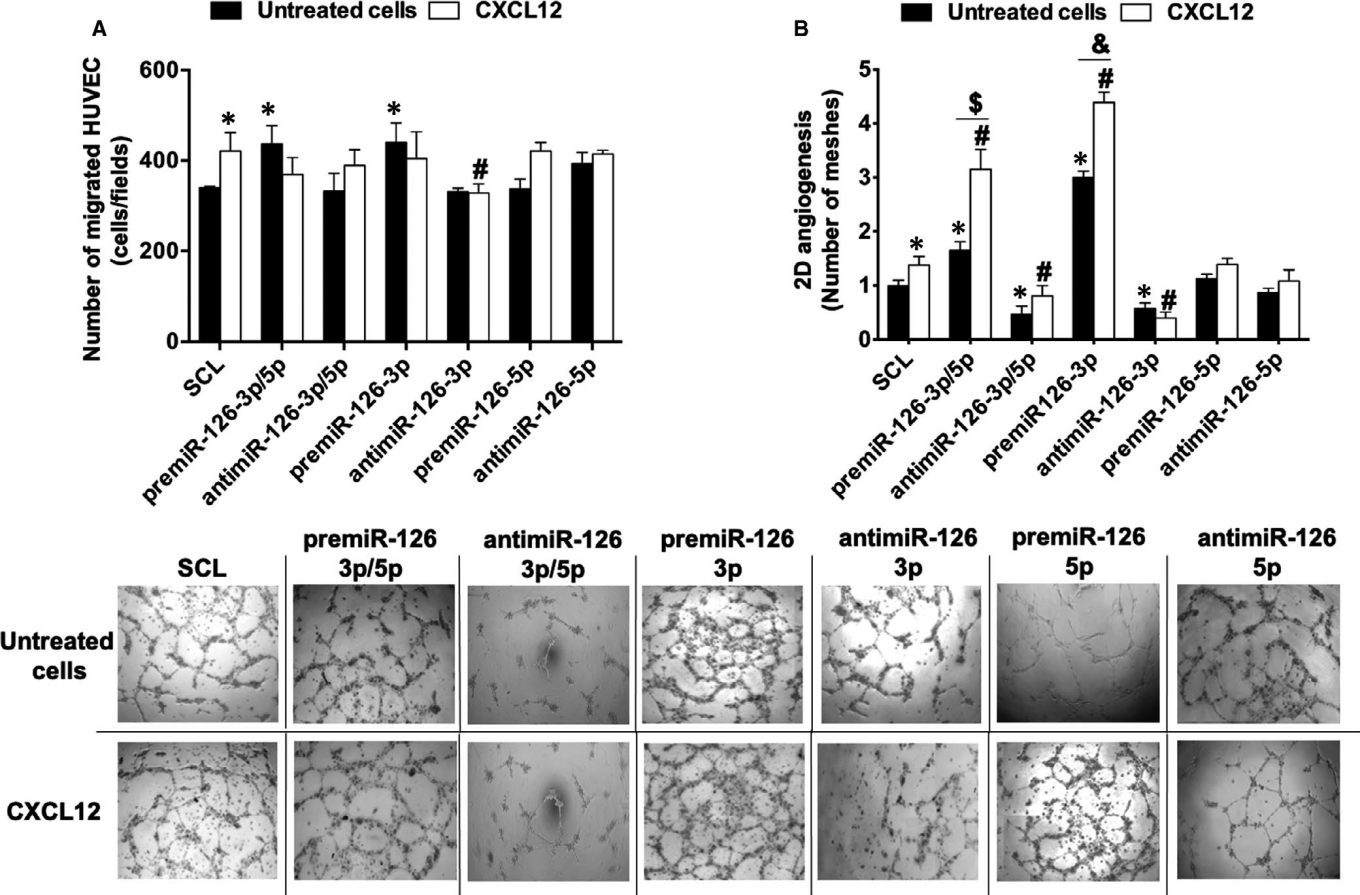
**Effect of miR‐126 on CXCL12‐induced migration and vascular tubes formation in vitro**. For migration assay (A), 5.10^4^ transfected HUVEC were deposited on the upper chamber of Boyden chamber and migration was stimulated by adding CXCL12 in the bottom chamber at 6 nmol.L^‐1^ for 24 h. Quantification of migrated cells was performed using phase contrast microscope. For vascular tubes formation in vitro (B), 7500 transfected HUVEC were deposited on Matrigel surface and stimulated during 6 h with CXCL12 at 6 nmol.L^‐1^; quantification of the number of meshes was performed using phase contrast microscope and Archimed^(TM)^ and Histolab^(TM)^ software. The results are expressed with mean ± SEM. Three independent experiments were performed for each assay. *p <.05 vs SCL; ^#^ *p* <.05 vs SCL + CLXC12; ^$^ *p* <.05 premiR‐126‐3p/5p vs premiR‐126‐3p/5p + CXCL12; ^&^ *p* <.05 premiR‐126‐3p vs premiR‐126‐3p + CXCL12

The next step of our study was to analyse the impact of miR‐126 deregulation on HUVEC migration, induced by CXCL12. Our results indicated (Figure 3A) a significant increase of HUVEC migration up to 24 ± 16%, when the cells were stimulated by CXCL12 as compared to SCL. Moreover, there was a significant decrease of HUVEC migration up to 22 ± 8% after antimiR‐126‐3p transfection and CXCL12 stimulation as compared to stimulation with CXCL12 only. In addition, the results showed that there was a significant increase of cell migration up to 25 ± 7% after premiR‐126‐5p transfection and CXCL12 stimulation as compared to the transfection with premiR‐126‐5p only. In contrast, there was no effect on HUVEC migration after antimiR‐126‐5p, premiR‐126‐3p or antimiR‐126‐3p/5p transfection and CXCL12 stimulation.

Our data suggest that miR‐126‐3p, but not miR‐126‐5p, was necessary in CXCL12‐induced HUVEC migration.

### 3.4 miR‐126‐3p was required for CXCL12‐induced 2D‐angiogenesis in vitro

The results showed (Figure 3B) that there was a significant increase of 2D‐angiogenesis up to 38 ± 16% after CXCL12 stimulation as compared to SCL. In addition, there was a significant increase up to 2.3 ± 0.3‐fold of 2D‐angiogenesis after HUVEC premiR‐126‐3p/5p transfection and CXCL12 stimulation as compared to stimulation with CXCL12 only. Interestingly, there was a significant increase of 2D‐angiogenesis up to 91 ± 23% after premiR‐126‐3p/5p transfection and CXCL12 stimulation as compared to transfection with premiR‐126‐3p/5p only. Moreover, there was a significant decrease in 2D‐angiogenesis up to 42 ± 14% after antimiR‐126‐3p/5p transfection and CXCL12 stimulation as compared to stimulation with CXCL12 only, or to transfection with antimiR‐126‐3p/5p only.

Then, we studied the role of miR‐126‐3p and miR‐126‐5p separately in HUVEC 2D‐angiogenesis. Our results showed (Figure 3B) that there was a significant increase of this effect up to 3.2 ± 0.2‐fold after premiR‐126‐3p transfection and CXCL12 stimulation as compared to stimulation with CXCL12 only. We also demonstrated a significant increase of 46 ± 6% as compared to transfection with premiR‐126‐3p only (Figure 3B). Interestingly, the results showed that there was a significant decrease in 2D‐angiogenesis up to 71 ± 8% after antimiR‐126‐3p transfection and CXCL12 stimulation as compared to stimulation with CXCL12 only. There were no significant changes as compared to transfection with antimiR‐126‐3p only. However, there were no significant changes in 2D‐angiogenesis after modulation of miR‐126‐5p expression.

Taken together, our results suggest that miR‐126‐3p/5p, miR‐126‐3p but not miR‐126‐5p are necessary in CXCL12‐induced HUVEC 2D‐angiogenesis. Moreover, miR‐126‐3p/5p and miR‐126‐3p potentialized CXCL12‐induced angiogenesis in vitro.

### 3.5 miR‐126 was implicated in CXCL12‐induced angiogenesis in an ex vivo rat aorta model

Herein, we investigated the miR‐126 involvement in CXCL12‐induced ex vivo angiogenesis by analysing the number of meshes formed on Matrigel layer. For this, the rat aortic rings were placed on Matrigel, transfected by miRs and then stimulated by CXCL12.

**FIGURE 4:**
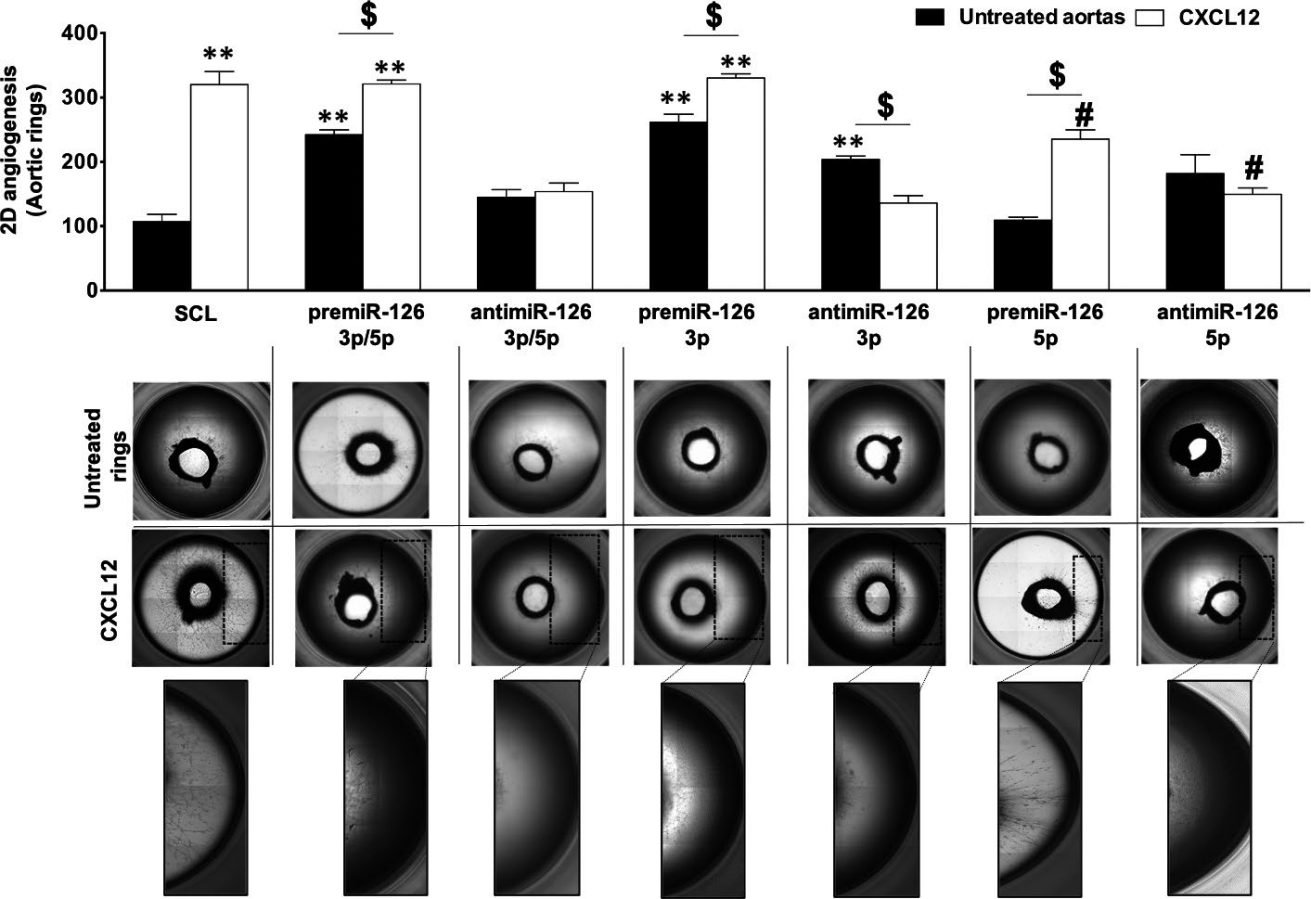
**Effect of miR‐126 on CXCL12‐induced vascular tubes formation ex vivo**. To analyse the implication of miR‐126 on CXCL12‐induced angiogenesis ex vivo, the aortic rings from Sprague‐Dawley Rats were transfected and stimulated with CXCL12 at 6 nmol.L*^‐1^* for 5 d. Quantification of the number of meshes was performed using phase contrast microscope and Archimed^(TM)^ and Histolab^(TM)^ software. The results are expressed with mean ± SEM. Five independent experiments were performed for each assay. ^#^ *p* <.05 vs SCL + CXCL12; **p <.05 vs SCL; ^$^ *p* <.05

First, we performed the analysis of CXCL12 and miRs separately on ex vivo angiogenesis. Our results showed (Figure 4) that there was a significant increase of angiogenesis up to 3 ± 0.3‐fold after CXCL12 stimulation (Figure 4). Moreover without CXCL12 stimulation, there was a significant increase in ex vivo angiogenesis up to 2.3 ± 0.1‐fold and 40 ± 10% after premiR‐126‐3p/5p or antimiR‐126‐3p/5p transfection respectively. Then, we compared the effects of miR‐126‐3p and miR‐126‐5p strands separately. The results showed a significant increase of angiogenesis up to 2.4 ± 0.2‐fold or 90 ± 10% or 70 ± 40% after premiR‐126‐3p or antimiR‐126‐3p or antimiR‐126‐5p transfection, respectively. There were no significant changes after premiR‐126‐5p transfection.

Second, we analysed the effect of CXCL12's angiogenesis stimulation after ex vivo miRs‐ transfection of aortas ring. Our results indicated a significant increase of angiogenesis up to 33 ± 4% after premiR‐126‐3p/5p transfection and CXCL12 stimulation as compared to premiR‐126‐3p/5p alone. However, there were no significant changes after premiR‐126‐3p/5p transfection and CXCL12 stimulation as compared to CXCL12 alone. Interestingly, the results showed that there was a significant decrease of angiogenesis up to 52 ± 6% after antimiR‐126‐3p/5p transfection and CXCL12 stimulation as compared to CXCL12 alone. In addition, there were no significant differences after antimiR‐126‐3p/5p transfection and CXCL12 stimulation as compared to antimiR‐126‐3p/5p alone.

Finally, we studied the implication of miR‐126 on ex vivo angiogenesis using separately the miR‐126‐3p and miR‐126‐5p strands. For the −3p strand, the results showed that there was a significant increase of ex vivo angiogenesis up to 26 ± 4% after premiR‐126‐3p transfection and CXCL12 stimulation as compared to premiR‐126‐3p alone. However, there were no significant changes between aortas transfected with premiR‐126‐3p and stimulated with CXCL12 as compared to CXCL12 alone. Interestingly, the results showed that there was a significant decrease of angiogenesis up to 57.5 ± 5% after antimiR‐126‐3p transfection and CXCL12 stimulation as compared to CXCL12 alone. In addition, there was a significant decrease up to 33 ± 8% after antimiR‐126‐3p transfection and stimulation by CXCL12 as compared to antimiR‐126‐3p alone.

Furthermore, for the −5p strand, the results showed that there was a significant decrease of angiogenesis up to 26 ± 6% after premiR‐126‐5p transfection and CXCL12 stimulation as compared to CXCL12 alone. Interestingly, there was a significant increase up to 2.2 ± 0.2‐fold after premiR‐126‐5p transfection and CXCL12 stimulation as compared to premiR‐126‐5p alone. Finally, the results showed that there was a significant decrease in angiogenesis up to 53 ± 4% after antimiR‐126‐5p transfection and CXCL12 stimulation as compared to CXCL12 alone. However, there were no significant changes between aortas transfected with antimiR‐126‐5p and stimulated with CXCL12 with those transfected only with antimiR‐126‐5p.

Our result suggests that miR‐126‐3p/5p and miR‐126‐3p but not miR‐126‐5p were necessary for CXCL12‐induced angiogenesis ex vivo. In contrast, modulation (up or downregulation) of miR‐126‐5p seems to alter CXCL12 angiogenic properties ex vivo.

### 3.6 SPRED‐1 inhibition induced 2D‐angiogenesis in vitro

**FIGURE 5:**
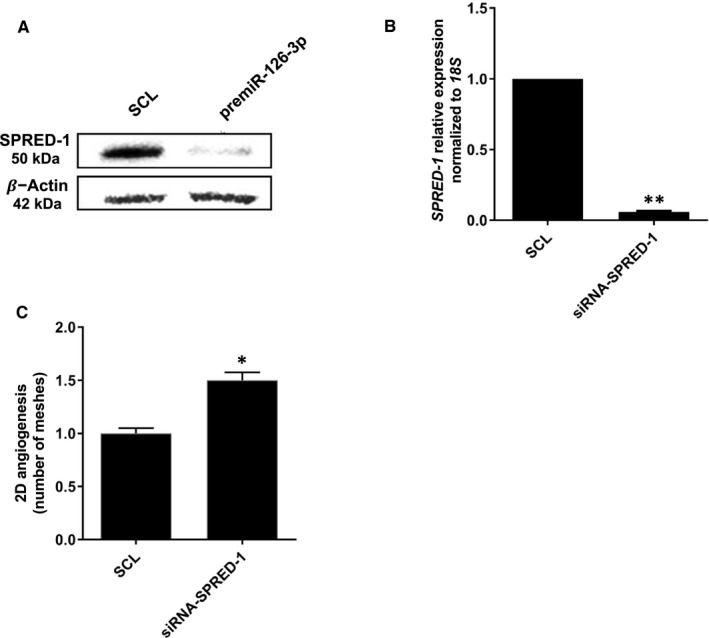
**SPRED‐1 is implicated in CXCL12/miR‐126‐induced vascular tubes formation**. (A) To determine the effect of miR‐126‐3p on SPRED‐1 expression, HUVEC were transfected with pre‐miR‐126‐3p for 24 h. Total proteins were extracted, and Western blot analysis was performed. (B) The inhibition of SPRED‐1 mRNA was checked by qRT‐PCR and using 18S rRNA as endogenous control (C) To analyse the implication of SPRED‐1 on CXCL12 induced vascular tubes formation in vitro, HUVEC were transected with siRNA‐SPRED‐1 at 25 nmol.L^‐1^ during 24 h. 7000 HUVECs were seeded on Matrigel and stimulated by CXCL12 at 6 nmol.L^‐1^ for 6 h. Vascular tubes formation was observed and the quantity of meshes was determined using phase contrast microscope and Archimed^(TM)^ and Histolab^(TM)^ software. Data were presented as the mean ± SEM of three and four independent experiments. **p <.01 vs Untreated cells; *p <.05 vs Untreated aortas

Since the −5p species had no significant effects in our previous models, we decided to focus on the −3p species for the rest of the study. To determine the essential role of miR‐126‐3p in CXCL12‐induced angiogenesis, we focused on SPRED‐1 expression, since SPRED‐1 is a known target of miR‐126‐3p. First, we confirmed that there was a significant decrease of SPRED‐1 protein level after premiR‐126‐3p transfection in HUVEC (Figure 5A). We hypothesized that its downregulation is necessary for CXCL12‐induced angiogenesis. To study this, we decided to analyse 2D‐angiogenesis in HUVEC in the presence of siRNA‐SPRED‐1 after CXCL12 stimulation. We validated the siRNA‐SPRED‐1 efficiency showing the abolition of SPRED‐1 mRNA expression compared to SCL (Figure 5B). Second, we analysed 2D‐angiogenesis in HUVEC after siRNA‐SPRED‐1 transfection. The results showed that there was a significant increase of 2D‐angiogenesis up to 50 ± 15% after siRNA‐SPRED‐1 transfection as compared to HUVEC transfected with SCL (Figure 5C).

Finally, our results suggest that SPRED‐1 is a negative regulator of HUVEC’s 2D‐angiogenesis.

### 3.7 CXCL12 decreased SPRED‐1 expression leading to increase of 2D‐angiogenesis

In the last part of this study, we wanted to know if downregulation of SPRED‐1 by miR‐126‐3p is implicated in CXCL12‐dependent angiogenesis.

**FIGURE 6:**
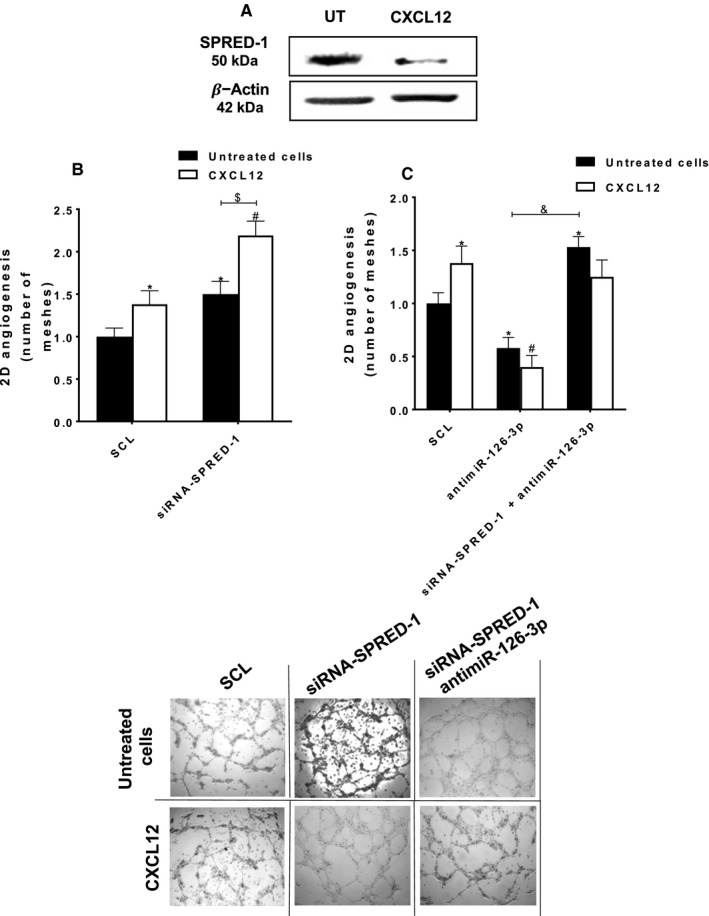
**miR‐126‐3p is implicated in the pro‐angiogenic effect of SPRED‐1 abolition**. (A) To analyse the effect of CXCL12 on SPRED‐1 expression, the HUVEC were stimulated or not with CXCL12 (6 nmol.L^‐1^) for 24 h. Total proteins were extracted, and Western blot analysis was performed. (B) To analyse the implication of SPRED‐1 in CXCL12‐induced angiogenesis, HUVEC were transfected with siRNA‐SPRED‐1 (25 nmol.L^‐1^), deposited on Matrigel and stimulated with CXCL12 (6 nmol.L^‐1^) for 6 h. (C) To analyse the implication of miR‐126‐3p in the pro‐angiogenic effect of the abolition of SPRED‐1, HUVEC and were co‐transfected with siRNA‐SPRED‐1 (25 nmol.L^‐1^) and antimiR‐126‐3p (20 nmol.L^‐1^). After co‐transfection, HUVEC were deposited on Matrigel and stimulated by CXCL12 (6 nmol.L^‐1^) for 6 h. For all analysis of vascular tubes formation and meshes quantification, the phase contrast microscope and the Archimed^(TM)^ and the Histolab^(TM)^ software were used. The results are expressed as mean ± SEM of three independent experiments. *p <.05 vs SCL; ^#^ *p* <.01 vs SCL + CXCL12; ^$^ *p* <.05 siRNA‐SPRED‐1 vs siRNA‐SPRED‐1 + CXCL12; ^&^ *p* <.05 antimiR‐126‐3p vs antimiR‐126‐3p + siRNA‐SPRED‐1. Magnification: x40

Since we proved that CXCL12 increased miR‐126‐3p level in HUVEC, we hypothesized that CXCL12 can modulate the SPRED‐1 expression. For the first time, the results showed that CXCL12 triggered a significant decrease of SPRED‐1 protein level at 24 hours of treatment (Figure 6A).

Interestingly, the results showed that siRNA‐SPRED‐1 transfection and CXCL12 stimulation led to a significant increase of 2D‐angiogenesis up to 59 ± 9% as compared to CXCL12 alone (Figure 6B, white arrows).

Then, we compared the effect of both siRNA‐SPRED‐1 and antimiR‐126‐3p co‐transfection on HUVEC 2D‐angiogenesis with transfection of siRNA‐SPRED‐1 only. The results showed that there was a significant increase of 2D‐angiogenesis up to 2.6 ± 0.1‐fold after siRNA‐SPRED‐1 and antimiR‐126‐3p co‐transfection as compared to transfection with antimiR‐126‐3p only (Figure 6C, black arrows). Interestingly, CXCL12 addition had no pro‐angiogenic effect on co‐transfected HUVEC (siRNA‐SPRED‐1 and antimiR‐126‐3p).

Overall, these results demonstrated that inhibition of SPRED‐1 potentializes CXCL12 pro‐angiogenic effect. Moreover miR‐126‐3p was crucial for CXCL12‐induced angiogenesis.

Taken together our results showed that, in the absence of both SPRED‐1 (siRNA‐SPRED‐1) and miR‐126‐3p (antimiR‐126‐3p), CXCL12 alone had no pro‐angiogenic effect on EC.

In conclusion, these results suggested that the pro‐angiogenic effect of CXCL12 was dependent on miR‐126‐3p and an alternative signalling pathway parallel to SPRED‐1. Despite the potentiating effect of the absence of SPRED‐1, the presence of miR‐126‐3p was essential for the pro‐angiogenic effect of CXCL12.

## 4 DISCUSSION

Angiogenesis is a physiological process, necessary for cardiovascular disorders regeneration, particularly after ischemic injuries. In this context, the pro‐angiogenic factors such as chemokines and miRs can be implicated to stimulate angiogenesis.^22^ In this study, we decided to focus on CXCL12 and its effect on EC through miRs modulation. Among miRs, miR‐126, strongly expressed by EC, has been identified as a pro‐angiogenic factor, which acts by decreasing SPRED‐1 level and stimulating the Erk1/2 signalling pathway.^17^ Like most miRs, miR‐126 is produced from a double stranded duplex precursor that imbeds miR‐126‐3p and miR‐126‐5p complementary strands.^23^ Depending on the tissue or cell type, the guide and passenger strands of a miR can act in synergy or as antagonists to regulate various biological processes.^13^ For example, although they have different mRNA targets, miR‐30‐3p and miR‐30‐5p or miR‐145‐3p and miR‐145‐5p act in synergy in the tumour progression of glioma or bladder tumour cells.^24^, ^25^ Conversely, depending on their rate and location, miR‐155‐3p and miR‐155‐5p may act together or against each other in dendritic and astrocytic cells.^26^, ^27^ Therefore, depending on the tissue environment and pathophysiological conditions, the two strands (−3p or −5p) of the same miR may have different roles and this requires studying them simultaneously in order to know the involvement of each strands in a pathophysiological process. This is why we wanted to study the role of both strands (miR‐126‐3p and miR‐126‐5p) in angiogenesis. These roles have never been compared at the same time and in the same experimental conditions in this physiological process.

Recently, it has been shown that the pro‐angiogenic process is controlled by chemokines through miRs regulation in chondrosarcoma and osteosarcoma cells.^28^, ^29^, ^30^, ^31^ However, there is no data indicating how chemokines impact miR regulation in the EC biological response and their impact on chemokine‐induced angiogenesis.

The aim of our work was to compare, in parallel, the effect of separate and combined miR‐126‐3p and miR‐126‐5p action on EC migration and on vascular tubes formation (2D‐angiogenesis) induced by CXCL12 and mediated by EC.

Our results showed for the first time that CXCL12 increased miR‐126‐3p expression, in both HUVEC in vitro and rat aortas ex vivo models and that this effect was associated with activation of *egfl7*‐miR‐126 promoter. Knowing that miR‐126‐3p is encoded by the 7th intron of the *egfl7* gene, we wished to demonstrate an increase of the promoter transcriptional activity using a plasmid reporter strategy associated with luciferase. Since the HUVEC lipofection leads to very low transfection efficiency and based on previous reports,^17^, ^32^, ^33^, ^34^, ^35^ we chose the human hepatocyte‐derived carcinoma cell line Huh7 to study this promoter activity. Although they represent a different cell model, it has been shown that the Huh7 cells present the CXCL12 specific receptors on their surface and, CXCL12 induce the similar signalling pathways (MAPK Erk1/2 and PI3K/Akt) that we found in HUVEC.^36^ In this context, we demonstrated that CXCL12 stimulation was associated with the activation of *egfl7*‐miR‐126 promoter. Since Ets1/2 is known as a specific transcription factor for *egfl7*,^37^ we could hypothesize that CXCL12 induced *egfl7* transcriptional activity through an increase of Ets1/2 (Figure S1). The e*gfl7* transcription start site contains 2 Ets binding sites that bind Ets1/2 transcription factor.^17^ Mutation of the Ets binding element decreases promoter transactivation and decreases miR‐126 expression.^37^ It has been previously shown that different growth factors and chemokines activate the Ets transcription factor, for example: CXCL12‐induced colorectal cancer cells migration via upregulation of Ets1.^38^ Based on these studies and in light of our results we hypothesize that CXCL12 enhances the miR‐126 promotor activity by the upregulation of the Ets transcription factor responsible of miR‐126 expression.

Regarding angiogenesis stimulation, according to the literature, CXCL12 and miR‐126‐3p had pro‐angiogenic effect on both migration and 2D‐angiogenesis in vitro and ex vivo^39^, ^40^ models. However, in our experimental conditions the miR‐126‐5p did not seem to have any detectable effect on this process.

Our results and others^17^ suggest that miR‐126‐3p had a strong pro‐angiogenic potential. Our model showed that miR‐126‐5p alone had no effect on 2D‐angiogenesis. However, its over‐expression (after premiR‐126‐3p/5p co‐transfection) reduced the pro‐angiogenic effect of miR‐126‐3p in HUVEC. In contrast, Zhou et al,^41^ found in retinal EC, that silencing the miR‐126‐3p repressed angiogenesis, while the over‐expression of miR‐126‐5p increased angiogenesis.^41^

We believe that this discrepancy was due to the differences between the experimental models and experimental conditions (cells types, presence of growth factors, and time of vascular tubes formation).

We further demonstrated that miR‐126‐3p is crucial for CXCL12‐induced migration and 2D‐angiogenesis in both in vitro and ex vivo models. Indeed, we showed that in absence of miR‐126‐3p (after anti‐miR‐126‐3p transfection) there was an abolition of CXCL12 pro‐angiogenic properties. These data suggest that the presence of miR‐126‐3p is essential to stimulate the pro‐angiogenic pathways induced by CXCL12. However, we observed conflicting results in our 2D‐angiogenesis ex vivo model. Indeed, in the absence of CXCL12 stimulation, we observed that the inhibition of miR‐126‐3p leads to an increase of 2D‐angiogenesis ex vivo. Interestingly, we and others have previously shown that the absence of miR‐126‐3p leads to CXCL12 synthesis and secretion in HUVEC culture medium.^21^, ^42^

In addition, since in our ex vivo experimental condition the miR transfection was done into the whole aorta, not only the EC but also the smooth muscle cells (SMC) and the fibroblasts could be transfected. In this context, it has been demonstrated by Jansen et al,^43^ that inhibition of miR‐126‐3p in SMC leads to an increase of its proliferation. Furthermore, it has been shown that the absence of miR‐126‐3p can lead to VEGF‐A synthesis.^44^

Since CXCL12 and antimiR‐126‐3p have been previously shown to enhance VEGF‐A expression^44^, ^45^, ^46^ and both of them can stimulate PKC/Erk1/2 pro‐angiogenic pathways through the stimulation of Raf protein,^47^, ^48^, ^49^ we hypothesize that antimiR‐126‐3p could have pro‐angiogenic action in our long‐term ex vivo tissue culture model. In addition, since in ex vivo experiments the rat aortas were kept in ex vivo tissue culture for 9 days, we hypothesized that after antimiR‐126 transfection there was an increase of SMC proliferation associated with VEGF synthesis, which could explain the pro‐angiogenic effect in the absence of CXCL12. However, in the presence of CXCL12 we showed that miR‐126‐3p was crucial for the chemokine pro‐angiogenic effects.

Then, we hypothesized that SPRED‐1 (a miR‐126‐3p known target)^17^ could be implicated in CXCL12‐induced angiogenesis. To prove this, we showed for the first time that CXCL12 inhibited SPRED‐1 expression in HUVEC.

Interestingly, our results showed that, the knock‐down of SPRED‐1 expression led to the stimulation of 2D‐angiogenesis in HUVEC associated with an increase in CXCL12‐induced angiogenesis. These data suggest a relationship between SPRED‐1 and CXCL12. Knowing that, we hypothesized that inhibition of SPRED‐1 could abolish the anti‐angiogenic effect induced by the absence of miR‐126‐3p and restored with CXCL12 pro‐angiogenic function. We demonstrated, in accordance with Wang et al,^17^ that the knock‐down of SPRED‐1 expression blocked the action of antimiR‐126‐3p leading to inhibition of the antimiR‐126‐3p‐based decrease of angiogenesis. However, in the presence of CXCL12, although inhibition of SPRED‐1 abolished the antimiR‐126‐3p‐based decrease of angiogenesis, this effect was not sufficient to recover an equivalent level to that which was observed after the transfection of siRNA‐SPRED‐1 only.

As demonstrated by Ho et al,^50^ in order to show the CXCL12 pro‐angiogenic effects, this process requires the joint activation of the Erk1/2 and PI3K/Akt signalling pathways. Furthermore, in EC, SPRED‐1 acts as a negative regulator of the Erk1/2 signalling pathway. Under our experimental conditions, although the Erk1/2 pathway can be unblocked by the SPRED‐1 inhibition, it has been demonstrated by Fish et al,^11^ that miR‐126‐3p inhibition leads to the PI3K/Akt‐pathway inactivation through the PI3KR2 modulation (Figure S2). Thus, in our model, when SPRED‐1 and miR‐126‐3p were inhibited (after co‐transfection with siRNA‐SPRED‐1 and antimiR‐126‐3p), the Erk1/2 pathway could be activated while the PI3K/Akt channel remains inactivated. Therefore, the inhibition of SPRED‐1 (after siRNA‐SPRED‐1 transfection) was not sufficient to restore the CXCL12 pro‐angiogenic effects in antimiR‐126‐transfected HUVEC.

**FIGURE 7:**
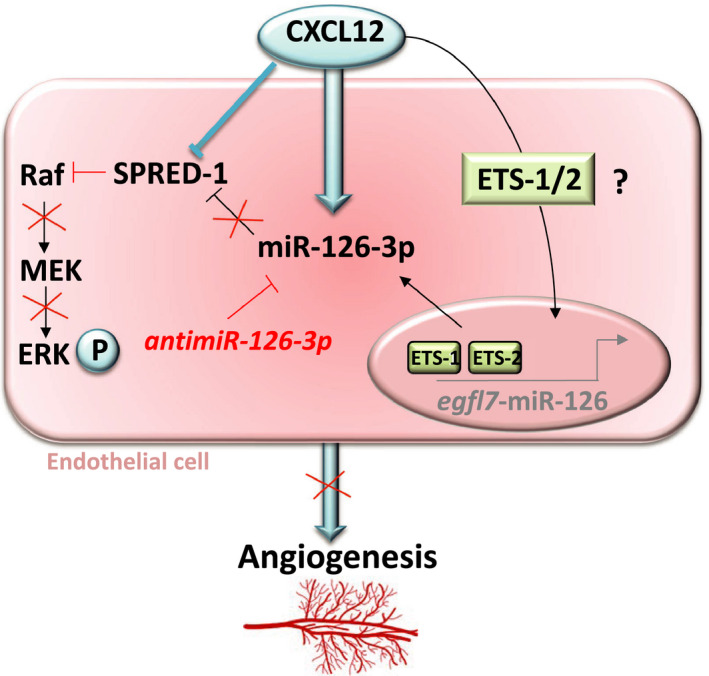
**CXCL12 induces angiogenesis through miR‐126‐3p/SPRED‐1 stimulation**. Our results showed for the first time that CXCL12 enhance miR‐126‐3p expression and its inhibition leads to a decrease of angiogenesis induced by CXCL12 in vitro. Moreover CXCL12 induced a decrease in SPRED‐1 (miR‐126‐3p known target) and this downregulation improves CXCL12‐induced angiogenesis in vitro. In this context, we hypothesized that CXCL12 induced miR‐126‐3p expression through the expression of Ets1/2 transcription factor complex

Taken together, under these conditions, it is clear that miR‐126‐3p plays a key role in CXCL12‐induced activation of both Erk1/2 and PI3K/Akt pro‐angiogenic pathways (Figure 7).

## 5 CONCLUSION

In conclusion, in this study we focused on two pro‐angiogenic factors, the miR‐126 and chemokine CXCL12, showing that the miR‐126/CXCL12 axis was implicated in endothelial cell migration and vascular tubes formation. In this context, we demonstrated that: (a) CXCL12 modulated the miR‐126‐3p expression in vitro in HUVEC model, as well as ex vivo in rat aortas model; (b) miR‐126 was necessary for the pro‐angiogenic effect induced by CXCL12; (c) SPRED‐1 was implicated in CXCL12‐induced angiogenesis; (d) miR‐126‐3p enhanced cell migration and vascular tubes formation, in HUVEC, however, the miR‐126‐5p had no effect on both processes.

## CONFLICT OF INTEREST

The author declares that there is no conflict of interest.

## AUTHOR CONTRIBUTIONS

**Kévin BASSAND:** Conceptualization (lead); Formal analysis (lead); Funding acquisition (equal); Investigation (lead); Methodology (lead); Project administration (lead); Supervision (equal); Validation (lead); Visualization (lead); Writing‐original draft (lead); Writing‐review & editing (lead). **Laurent Metzinger:** Conceptualization (equal); Methodology (equal); Writing‐original draft (equal). **Meriem NAIM:** Writing‐original draft (supporting); Writing‐review & editing (equal). **Nesrine MOUHOUBI:** Conceptualization (supporting). **Oualid Haddad:** Conceptualization (supporting); Investigation (supporting); Methodology (supporting); Resources (supporting). **Vincent ASSOUN:** Investigation (supporting). **Naima ZAIDI:** Resources (supporting). **Odile SAINTE‐CATHERINE:** Investigation (supporting); Resources (supporting). **Amena BUTT:** Conceptualization (supporting); Resources (supporting). **Erwan GUYOT:** Conceptualization (supporting); Funding acquisition (equal). **Olivier OUDAR:** Conceptualization (supporting). **Christelle Laguillier‐Morizot:** Conceptualization (supporting); Funding acquisition (equal). **Angela SUTTON:** Conceptualization (supporting); Funding acquisition (equal); Writing‐original draft (supporting); Writing‐review & editing (supporting). **Nathalie CHARNAUX:** Conceptualization (supporting). **Valérie METZINGER‐LE MEUTH:** Conceptualization (equal); Methodology (equal); Writing‐original draft (equal). **Hanna HLAWATY:** Conceptualization (lead); Formal analysis (lead); Funding acquisition (lead); Methodology (lead); Project administration (lead); Resources (equal); Supervision (lead); Validation (lead); Visualization (lead); Writing‐original draft (lead); Writing‐review & editing (lead).

## Supporting information

Fig S1‐S2

## Data Availability

Data supporting the findings of this study could be obtained from the corresponding author upon reasonable request.

## References

[1] Poole TJ , Finkelstein EB , Cox CM . The role of FGF and VEGF in angioblast induction and migration during vascular development. Dev Dyn. 2001;220(1):1‐17. 11146503 10.1002/1097-0177(2000)9999:9999<::AID-DVDY1087>3.0.CO;2-2

[2] Zhang Y , Zhang H , Lin S , et al. SDF‐1/CXCR7 chemokine signalling is induced in the peri‐infarct regions in patients with ischemic stroke. Aging Dis. 2018;9(2):287‐295. 29896417 10.14336/AD.2017.1112 PMC5963349

[3] Yamagami S , Tamura M , Hayashi M , et al. Differential production of MCP‐1 and cytokine‐induced neutrophil chemoattractant in the ischemic brain after transient focal ischemia in rats. J Leucok Biol. 1999;65(6):744‐749. 10.1002/jlb.65.6.744 10380894

[4] Suffee N , Hlawaty H , Meddahi‐Pelle A , et al. RANTES/CCL5‐induced pro‐angiogenic effects depend on CCR1, CCR5 and glycosaminoglycans. Angiogenesis. 2012;15(4):727‐744. 22752444 10.1007/s10456-012-9285-x

[5] Mehrad B , Keane MP , Strieter RM . Chemokines as mediators of angiogenesis. Thromb Haemost. 2007;97(5):755‐762. 17479186 PMC3353527

[6] Yin Y , Zhao X , Fang Y , Yu S , Zhao J . SDF‐1 α involved in mobilization and recruitment of endothelial progenitor cells after arterial injury in mice. Cardiovasc Pathol. 2010;19(4):218‐227. 19502087 10.1016/j.carpath.2009.04.002

[7] Ho TK , Tsui J , Xu S , Leoni P , Abraham DJ , Baker DM . Angiogenic effects of stromal cell‐derived factor‐1 (SDF‐1/CXCL12) variants in vitro and the in vivo expressions of CXCL12 variants and CXCR4 in human critical leg ischemia. J Vasc Surg. 2010;51(3):689‐699. 20206813 10.1016/j.jvs.2009.10.044

[8] Welten SMJ , Goossens EAC , Quax PHA , Nossent AY . The multifactorial nature of microRNAs in vascular remodelling. Cardiovasc Res. 2016;110(1):6‐22. 26912672 10.1093/cvr/cvw039

[9] Bartel DP , Chen C‐Z . Micromanagers of gene expression: the potentially widespread influence of metazoan microRNAs. Nat Rev Genet. 2004;5(5):396‐400. 15143321 10.1038/nrg1328

[10] Sun L‐L , Li W‐D , Lei F‐R , Li X‐Q . The regulatory role of microRNAs in angiogenesis‐related diseases. J Cell Mol Med. 2018;22(10):4568‐4587. 29956461 10.1111/jcmm.13700 PMC6156236

[11] Fish JE , Santoro MM , Morton SU , et al. miR‐126 regulates angiogenic signaling and vascular integrity. Dev Cell. 2008;15(2):272‐284. 18694566 10.1016/j.devcel.2008.07.008 PMC2604134

[12] Wang X , Lian Y , Wen X , et al. Expression of miR‐126 and its potential function in coronary artery disease. Afri Heal Sci. 2017;17(2):474‐480. 10.4314/ahs.v17i2.22 PMC5637033 29062343

[13] Meijer HA , Smith EM , Bushell M . Regulation of miRNA strand selection: follow the leader? Biochem Soc Trans. 2014;42(4):1135‐1140. 25110015 10.1042/BST20140142

[14] Poissonnier L , Villain G , Soncin F , Mattot V . MiR126‐5p repression of ALCAM and SetD5 in endothelial cells regulates leucocyte adhesion and transmigration. Cardiovasc Res. 2014;102(3):436‐447. 24562769 10.1093/cvr/cvu040

[15] Zhou Q , Anderson C , Hanus J , et al. Strand and cell type‐specific function of microRNA‐126 in. Angiogenesis. 2016;24(10):1823‐1835. 10.1038/mt.2016.108 PMC5112035 27203443

[16] Villain G , Poissonnier L , Noueihed B , et al. miR‐126‐5p promotes retinal endothelial cell survival through SetD5 regulation in neurons. Development. 2018;145(1):dev156232. 29180574 10.1242/dev.156232

[17] Wang S , Aurora AB , Johnson BA , et al. The endothelial‐specific microRNA miR‐126 governs vascular integrity and angiogenesis. Dev Cell. 2008;15(2):261‐271. 18694565 10.1016/j.devcel.2008.07.002 PMC2685763

[18] Quintanar‐Audelo M , Yusoff P , Sinniah S , Chandramouli S , Guy GR . Sprouty‐related Ena/vasodilator‐stimulated phosphoprotein homology 1‐domain‐containing protein (SPRED1), a tyrosine‐protein phosphatase non‐receptor type 11 (SHP2) substrate in the ras/extracellular signal‐regulated kinase (ERK) pathway. J Biol Chem. 2011;286(26):23102‐23112. 21531714 10.1074/jbc.M110.212662 PMC3123077

[19] Schober A , Nazari‐Jahantigh M , Wei Y , et al. MicroRNA‐126‐5p promotes endothelialproliferation and limits atherosclerosis by suppressing Dlk1. Nat Med. 2014;20(4):368‐376. 24584117 10.1038/nm.3487 PMC4398028

[20] Zernecke A , Bidzhekov K , Noels H , et al. Delivery of microRNA‐126 by apoptotic bodies induces CXCL12‐dependent vascular protection. Sci Signal. 2009;2(100):ra81. 19996457 10.1126/scisignal.2000610

[21] Mondadori dos Santos A , Metzinger L , Haddad O , et al. miR‐126 is involved in vascular remodeling under laminar shear stress. Biomed Res Int. 2015;2015:1‐11. 10.1155/2015/497280 PMC4499382 26221595

[22] Staszel T , Zapała B , Polus A , et al. Role of microRNAs in endothelial cell pathophysiology. Pol Arch Med Wewn. 2011;121(10):361‐367. 21946298

[23] Metzinger‐Le Meuth V , Burtey S , Maitrias P , Massy ZA , Metzinger L . microRNAs in the pathophysiology of CKD‐MBD: biomarkers and innovative drugs. Biochim BiophysActa ‐ Mol Basis Dis. 2017;1863(1):337‐345. 10.1016/j.bbadis.2016.10.027 27806914

[24] Jiang L , Lin C , Song L , et al. MicroRNA‐30e* promotes human glioma cell invasiveness in an orthotopic xenotransplantation model by disrupting the NF‐κ B/Iκ Bα negative feedback loop. J Clin Invest. 2012;122(1):33‐47. 22156201 10.1172/JCI58849 PMC3248293

[25] Matsushita R , Yoshino H , Enokida H , et al. Regulation of UHRF1 by dual‐strand tumor suppressor microRNA‐145 (miR‐145‐5p and miR‐145‐3p): inhibition of bladder cancer cell aggressiveness. Oncotarget. 2016;7(19):28460‐28487. 27072587 10.18632/oncotarget.8668 PMC5053739

[26] Zhou H , Huang X , Cui H , et al. miR‐155 and its star‐form partner miR‐155* cooperatively regulate type I interferon production by human plasmacytoid dendritic cells. Blood. 2010;116(26):5885‐5894. 20852130 10.1182/blood-2010-04-280156

[27] Tarassishin L , Loudig O , Bauman A , Shafit‐Zagardo B , Suh H‐S , Lee SC . Interferon regulatory factor 3 inhibits astrocyte inflammatory gene expression through suppression of the proinflammatory miR‐155 and miR‐155*. Glia. 2011;59(12):1911‐1922. 22170100 10.1002/glia.21233 PMC3241213

[28] Liu GT , Huang YL , Tzeng HE , Tsai CH , Wang SW , Tang CH . CCL5 promotes vascular endothelial growth factor expression and induces angiogenesis by down‐ regulating miR‐199a in human chondrosarcoma cells. Cancer Lett. 2015;357(2):476‐487. 25444917 10.1016/j.canlet.2014.11.015

[29] Liu G‐T , Chen H‐T , Tsou H‐K , et al. CCL5 promotes VEGF‐dependent angiogenesis by down‐regulating miR‐200b through PI3K/Akt signaling pathway in human. Oncotarget. 2014;5(21):10718‐10731. 25301739 10.18632/oncotarget.2532 PMC4279405

[30] Wang L‐H , Lin C‐Y , Liu S‐C , et al. CCL5 promotes VEGF‐C production and induces lymphangiogenesis by suppressing miR‐507 in human chondrosarcoma cells. Oncotarget. 2016;7(24):36896‐36908. 27166194 10.18632/oncotarget.9213 PMC5095047

[31] Liao Y‐Y , Tsai H‐C , Chou P‐Y , et al. CCL3 promotes angiogenesis by dysregulation ofmiR‐374b/ VEGF‐A axis in human osteosarcoma cells. Oncotarget. 2016;7(4):4310‐4325. 26713602 10.18632/oncotarget.6708 PMC4826207

[32] Zhou S , Liang P , Zhang P , Zhang M , Huang X . The long noncoding RNA PDK1‐AS/miR‐125b‐5p/VEGFA axis modulates human dermal microvascular endothelial cell and human umbilical vein endothelial cell angiogenesis after thermal injury. J Cell Physiol. 2020;236(4):3129‐3142. 33078418 10.1002/jcp.30081

[33] Pang J , Ye L , Chen Q , Wang J , Yang X , He W , Hao L . The effect of MicroRNA‐101 on angiogenesis of human umbilical vein endothelial cells during hypoxia and in mice with myocardial infarction. Biomed Res Int. 2020;2020:5426971. 32953883 10.1155/2020/5426971 PMC7487113

[34] Anene C , Graham AM , Boyne J , Roberts W . Platelet microparticle delivered microRNA‐Let‐7a promotes the angiogenic switch. Biochim Biophys Acta Mol Basis Dis. 2018;1864(8):2633‐2643. 29684582 10.1016/j.bbadis.2018.04.013

[35] Soufi‐Zomorrod M , Hajifathali A , Kouhkan F , Mehdizadeh M , Rad SM , Soleimani M . MicroRNAs modulating angiogenesis: miR‐129‐1 and miR‐133 act as angio‐miR in HUVECs. Tumour Biol. 2016;37(7):9527‐9534. 26790441 10.1007/s13277-016-4845-0

[36] Sutton A , Friand V , Brulé‐Donneger S , et al. Stromal cell–derived factor‐1/chemokine (C‐X‐C Motif) ligand 12 stimulates human hepatoma cell growth, migration, and invasion. Mol Cancer Res. 2007;5(1):21‐33. 17259344 10.1158/1541-7786.MCR-06-0103

[37] Harris Ta , Yamakuchi M , Kondo M , Oettgen P , Lowenstein CJ . Ets‐1 and Ets‐2 regulate the expression of miR‐126 in endothelial cells. Arterioscler Thromb Vasc Biol. 2010;30(10):1990‐1997. 20671229 10.1161/ATVBAHA.110.211706 PMC3121560

[38] Li P , Wei J , Li X , et al. 17β‐Estradiol enhances vascular endothelial Ets‐1/miR‐126‐3p expression: the possible mechanism for attenuation of atherosclerosis. J Clin Endocrinol Metab. 2017;102(2):594‐603. 27870587 10.1210/jc.2016-2974

[39] Wang Y , Huang J , Li Y , Yang G . Roles of chemokine CXCL12 and its receptors in ischemic. Stroke. 2012;13(2):166‐172. 10.2174/138945012799201603 22204316

[40] Qu Q , Bing W , Meng X , et al. Upregulation of miR‐126‐3p promotes human saphenous vein endothelial cell proliferation in vitro and prevents vein graft neointimal formation ex vivo and in vivo. Oncotarget. 2017;8(63):106790‐106806. 29290989 10.18632/oncotarget.22365 PMC5739774

[41] Zhou Q , Anderson C , Hanus J , et al. Strand and cell type‐specific function of microRNA‐126 in angiogenesis. Mol Ther. 2016;24(10):1823‐1835. 27203443 10.1038/mt.2016.108 PMC5112035

[42] Van Solingen C , De Boer HC , Bijkerk R , et al. MicroRNA‐126 modulates endothelial SDF‐1 expression and mobilization of Sca‐1+/Lin‐ progenitor cells in ischaemia. Cardiovasc Res. 2011;92(3):449‐455. 21856785 10.1093/cvr/cvr227

[43] Jansen F , Stumpf T , Proebsting S , et al. Intercellular transfer of miR‐126‐3p by endothelial microparticles reduces vascular smooth muscle cell proliferation and limits neointima formation by inhibiting LRP6. J Mol Cell Cardiol. 2017;104:43‐52. 28143713 10.1016/j.yjmcc.2016.12.005

[44] Ye P , Liu J , He F , Xu W , Yao K . Hypoxia‐induced deregulation of miR‐126 and its regulative effect on VEGF and MMP‐9 expression. Int J Med Sci. 2013;11(1):17‐23. 24396282 10.7150/ijms.7329 PMC3880987

[45] Ge HY , Han ZJ , Tian P , et al. VEGFA expression is inhibited by arsenic trioxide in HUVECs through the upregulation of Ets‐2 and miRNA‐126. PLoS One. 2015;10(8):1‐18. 10.1371/journal.pone.0135795 PMC4537190 26274316

[46] Salcedo R , Wasserman K , Young HA , et al. Vascular endothelial growth factor and basic fibroblast growth factor induce expression of CXCR4 on human endothelial cells in vivo neovascularization induced by. Stromal‐Derived. 1999;154(4):1125‐1135. 10.1016/s0002-9440(10)65365-5 PMC1866563 10233851

[47] Valdés G , Erices R , Chacón C , Corthorn J . Angiogenic, hyperpermeability and vasodilator network in utero‐placental units along pregnancy in the guinea‐pig (Cavia porcellus). Reprod Biol Endocrinol. 2008;6:1‐11. 18371207 10.1186/1477-7827-6-13 PMC2291058

[48] Cojoc M , Peitzsch C , Trautmann F , Polishchuk L , Telegeev GD , Dubrovska A . OTT‐36109‐emerging‐targets‐in‐cancer‐management–role‐of‐the‐cxcl12‐cx. Onco Targets Ther. 2013;6:1347‐1361. 24124379 10.2147/OTT.S36109 PMC3794844

[49] Moghaddam AB , Namvar F , Moniri M , Tahir PM , Azizi S , Mohamad R . Nanoparticlesbiosynthesized by fungi and yeast: a review of their preparation, properties, andmedical applications. Molecules. 2015;20(9):16540‐16565. 26378513 10.3390/molecules200916540 PMC6332129

[50] Ho TK , Shiwen X , Abraham D , Tsui J , Baker D . Stromal‐cell‐derived factor‐1 (SDF‐1)/CXCL12 as potential target of therapeutic angiogenesis in critical leg ischaemia. Cardiol Res Prac. 2012;2012:1‐7. 10.1155/2012/143209 PMC3296148 22462026

