# Supplementary material for: miR‐126‐3p is essential for CXCL12‐induced angiogenesis
Source: J Cell Mol Med. 2021 Jun 12;25(13):6032–45. doi: 10.1111/jcmm.16460 (PMC8256342; doi:10.1111/jcmm.16460)
Supplement: Supplementary file 1 — Fig S1‐S2 [file JCMM-25-6032-s001.pdf]

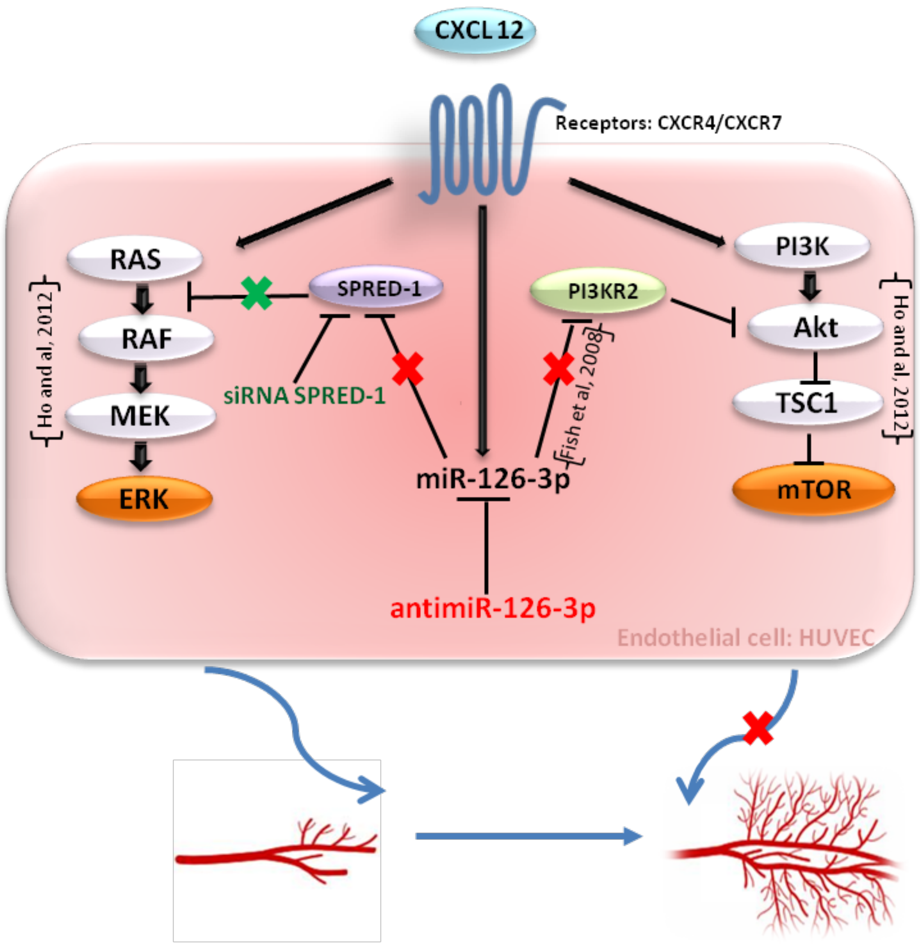

Supplemental Figure 1. Two parallel pathways implicated in angiogenesis: CXCL12 has a proangiogenic effect via the activation of Erk and Akt signaling pathways. *CXCL12 enhances miR-126-3p expression which inhibits SPRED-1 and PI3KR2, inducing the activation of Erk and Akt proangiogenic pathways.*

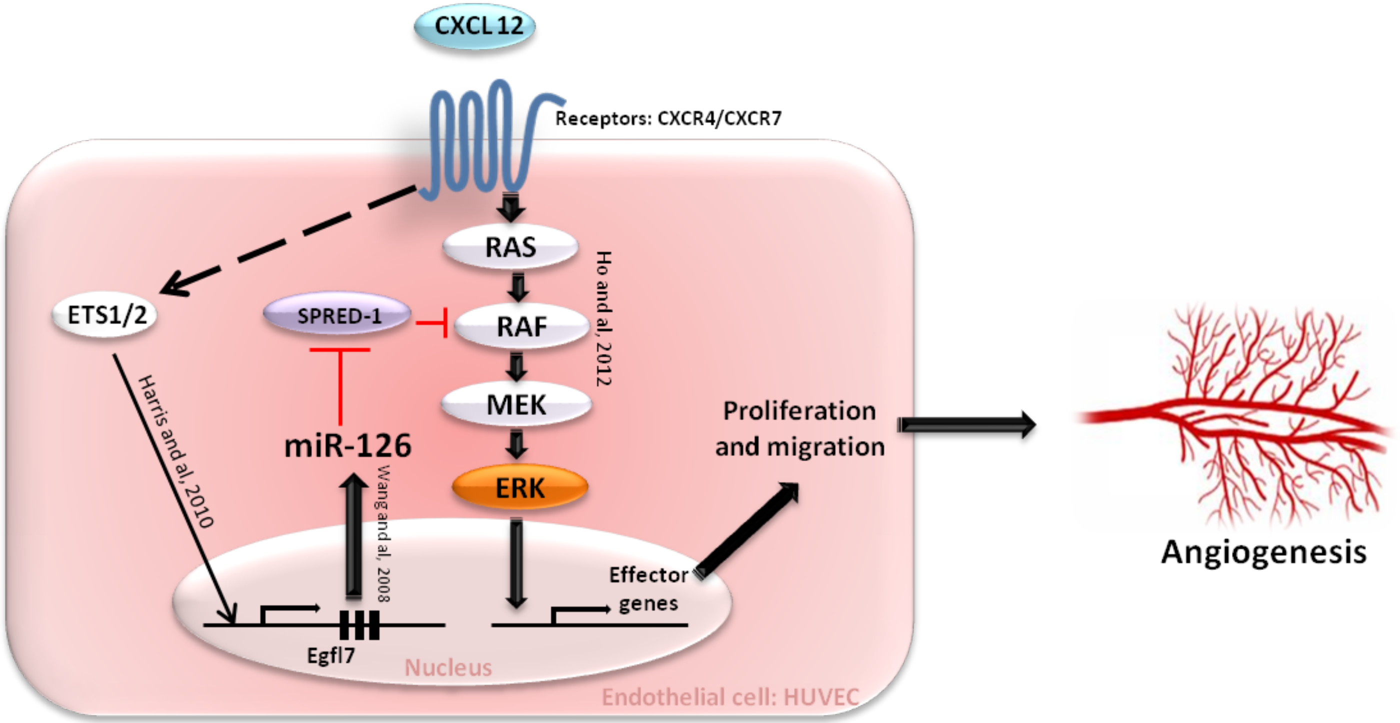

Supplemental Figure 2. Hypothetical representation of CXCL12-induced angiogenesis via increase of miR-126 expression through the enhancing of egfl7 promotor activity. *CXCL12-based treatment increased the miR-126 level, which inhibits SPRED-1 expression leading to activation of MAPK pathways and the indiuction of EC proliferation, migration and angiogenesis.*
